# Impact of fibrinogen level on the prognosis of patients with traumatic brain injury: a single-center analysis of 2570 patients

**DOI:** 10.1186/s13017-020-00332-1

**Published:** 2020-09-25

**Authors:** Ke Lv, Qiang Yuan, Pengfei Fu, Gang Wu, Xing Wu, Zhuoying Du, Jian Yu, Zhiqi Li, Jin Hu

**Affiliations:** Department of Neurosurgery, Huashan Hospital, Fudan University, 12 Wulumuqi Middle Road, Shanghai, China

**Keywords:** Traumatic brain injury, Fibrinogen, Coagulopathy, Mortality, Outcomes

## Abstract

**Background:**

Fibrinogen may play an important role in the survival of trauma patients; however, its role in traumatic brain injury (TBI) and its correlation with disease prognosis remain poorly understood. The aims of this study were to determine the incidence of TBI-associated hypofibrinogenemia in patients with TBI and to evaluate the prognostic value of fibrinogen level with respect to mortality and clinical outcomes.

**Methods:**

A total of 2570 consecutive TBI patients were retrospectively studied. Prognostic evaluations were determined using the Glasgow Outcome Score (GOS) assessment 3 months after injury. The shape of the relationship between fibrinogen level and mortality or outcome was examined using cubic spline functions. Logistic regression analyses were conducted to identify the association between fibrinogen level and 3-month functional outcomes.

**Results:**

Fibrinogen concentrations < 2 g/L were observed in 992 (38.6%) patients at the time of admission. Multivariate analyses showed that for patients with fibrinogen levels < 2.0 g/L, those levels were an independent prognostic factor for 3-month mortality (odds ratio [OR], 0.91; 95% confidence interval [CI], 0.89–0.93; *P* < .001). By contrast, for patients with fibrinogen levels < 2.5 g/L, the levels were an independent prognostic factor for favorable outcomes at 3 months (OR, 1.654; 95% CI, 1.186–2.306; *P* = .003). Similar results were also seen for patients with fibrinogen levels > 3.0 g/L, with the levels being an independent prognostic factor for favorable outcomes at 3 months (OR, 0.771; 95% CI, 0.607–0.979; *P* = .033).

**Conclusions:**

Fibrinogen is an independent prognostic factor for clinical outcomes in TBI patients. Maintaining the level of fibrinogen between 2.5 and 3 g/L may improve clinical outcomes in patients with TBI.

## Background

Traumatic brain injury (TBI) is a common factor contributing to neurological morbidity and the main cause of disability affecting patient function and quality of life [1]. Coagulopathies play an important role in the progression of intracranial hemorrhage and unfavorable outcomes after TBI [2]. Among the various factors affecting coagulation, fibrinogen is crucial for platelet aggregation, serving as the primary substrate for plasmatic coagulation, resulting in a mesh network used to enhance clot strength [3]. However, despite its importance, fibrinogen remains among the most vulnerable of the coagulation factors in patients sustaining serious injuries. In the early stages of trauma, fibrinogen consumption is increased, but its synthesis remains constant, resulting in critically low levels available [4, 5]. Beyond this rapid consumption, a variety of other causes help contribute to fibrinogen depletion following severe brain trauma, such as blood loss or dilution, consumption, hyperfibrinolysis, hypothermia, and acidosis, thereby further reducing its levels [6–8].

Hypofibrinogenemia is common in trauma patients, including 50–74% of trauma patients at the time of hospital admission, with levels below 2.29 g/L strongly associated with increased mortality [9, 10]. Juratli et al. reported that pathologic fibrinogen levels can be an independent risk factor for early progressive hemorrhagic injury in TBI patients [11]. In a recent clinical study, 71 of 141 TBI patients with hypofibrinogenemia showed progressive hemorrhagic injury in repeat head CT at 6 h (50%) [12]. In addition, low fibrinogen levels are associated with higher risk for elevated intracranial pressure in patients with severe TBI [13]. The European guidelines on management of major bleeding and coagulopathy following trauma recommend treatment with fibrinogen concentrate or cryoprecipitate if major bleeding is accompanied by hypofibrinogenemia [14]. Moreover, when fibrinogen levels in trauma patients fall below 2.29 g/L, every unit increase in fibrinogen concentration may reduce the mortality of trauma patients by 0.8% [10]. Although fibrinogen may play an important role in the survival of trauma patients, its role in TBI and its correlation with disease prognosis remain poorly understood [15]. Understanding the predictive ability of fibrinogen in TBI patients at admission will help in the early identification of at-risk patients, resulting in better management and improved prognosis [16].

The aims of our study were two-fold: first, to determine the incidence of TBI-associated hypofibrinogenemia in patients with TBI who were admitted to an adult neurotrauma center, and second, to evaluate the prognostic value of fibrinogen level with respect to mortality and clinical outcomes. To address these aims, we retrospectively investigated the relationship between the fibrinogen levels at the time of admission and clinical outcomes in TBI patients and assessed the optimal target level of fibrinogen which could potentially guide fibrinogen replacement for TBI patients.

## Materials and methods

### Patient population

A total of 2570 consecutive TBI patients admitted to the Neurotrauma Center between December 2005 and December 2019 were retrospectively studied. The inclusion criteria were as follows: TBI with radiological signs of intracranial brain injury (epidural or subdural hematoma [EDH or SDH], intraparenchymal hemorrhage [IPH], diffuse axonal injury [DAI] or contusion) documented via computed tomography (CT); ≥ 14 years of age; and admission within 24 h of TBI. Patients with penetrating brain injuries, preexisting coagulopathy, concurrent use of anticoagulant or antiplatelet agents, and preexisting severe hepatic insufficiency were excluded from the study. All patients were evaluated and treated according to the Guidelines for the Management of Severe Head Injury [17]. Multiple injuries are defined as patients with traumatic injury to a body region other than the brain with an Abbreviated Injury Severity score ≥ 3. For TBI patients with fibrinogen < 1.5 g/L, cryoprecipitate or fibrinogen were used to supplement the patient’s serum fibrinogen. For patients included retrospectively, an exemption from informed consent was approved by the local Privacy and Data Protection Officer.

### Data collection

Data obtained from TBI patients included age, sex, mechanism of injury, pupillary reaction to light, Injury Severity Score (ISS), Glasgow Coma Scale (GCS) at admission, CT findings, and site of multiple injury. In addition, initial CT scan results taken at the time of admission were used to assess the severity and type of injury. Platelet count and coagulation tests, including international normalized ratio (INR), prothrombin time (PT), and partial thromboplastin time (APTT), were performed, and fibrinogen (FIB) and D-dimer levels were measured, in all patients within 12 h of injury and assessed at the Central Clinical Chemistry Laboratory using routine laboratory assays. Hemoglobin (Hb) and hematocrit (HCT) levels were also measured and recorded.

The neurological outcomes were determined according to the Glasgow Outcome Score (GOS) as follows: 1 = dead; 2 = vegetative state with an inability to interact with the environment; 3 = severe disability with an inability to live independently but the ability to follow commands; 4 = moderate disability with the ability to live independently but an inability to return to work or school; and 5 = good recovery with the ability to return to work or school. Prognostic evaluations were determined using the GOS assessment at 3 months after injury. GOS evaluations were performed by physicians either in person or via telephone. A GOS of 1–3 was categorized as an unfavorable outcome, whereas a score of 4–5 was deemed a favorable outcome.

### Statistical analyses

Continuous variables are expressed as means ± standard deviations or medians (interquartile ranges), and categorical variables are expressed as percentages. Univariate analyses of categorical data were performed using the chi-square test. Equality of variance was assessed using Levene’s test. Normally distributed variables were compared using Student’s *t* test or analysis of variance, whereas non-normally distributed variables were compared using the Kruskal-Wallis test or Mann-Whitney *U* test. The shape of the relationship between FIB and mortality or outcome was examined using univariate analyses with linear and cubic spline functions. Following univariate analyses, forward stepwise logistic regression analyses were conducted to identify the association between fibrinogen levels and 3-month mortality or functional outcome. The R statistical package for Windows version 3.3.6 (The R Foundation for Statistical Computing) and SPSS Statistics (version 25.0.0; IBM Corp, Somers, NY, USA) were used to perform the statistical tests. *P* values < 0.05 were considered statistically significant. All *P* values were two-sided.

## Results

A total of 2570 hospitalized adult TBI patients were included in the analyses. The mean age of the patients studied was 48.54 ± 16.38 years, of which 1934 patients (73.5%) were male. The main cause of TBI was traffic accidents (*n* = 1465, 57.0%), followed by stumbles (*n* = 45217.6%) and falls (*n* = 378, 14.7%). Intraparenchymal hemorrhage and brain contusion were the most common head CT findings (*n* = 1840, 70.2%). Multiple injuries were seen in 147 (5.7%) patients. The most common site of multiple injuries was the thorax (*n* = 103, 4.0%), followed by the extremities (*n* = 37, 1.4%). The mean ISS of all patients was 15.35 ± 7.24. The mean fibrinogen level at admission was 2.54 ± 1.14 g/L, with 77 (3.0%) patients exhibiting fibrinogen levels < 1.0 g/L, 326 patients (12.7%) with levels between 1.0 and 1.5 g/L, 589 patients (22.9%) between 1.5 and 2.0 g/L, 918 patients (35.7%) between 2.0 and 3.0 g/L, and 660 patients (25.7%) > 3.0 g/L. A total of 198 patients died in this series. The main cause of death was brain death caused by malignant intracranial hypertension (*n* = 128, 64.6%), followed by multiple organ failure (*n* = 56, 28.3%), septic shock (*n* = 11, 5.6%) and intracranial infection (*n* = 3, 1.5%). Among them, 41.9% died within 24 h after admission, 24.2% died between 24 and 48 h, 25.8% died between 48 and 72 h, and 8.1% died after 72 h.

Univariate analyses revealed that age, pupillary reactions, GCS at admission, head CT findings, and ISS were significantly related to 3-month mortality. Meanwhile, patients who died exhibited significantly higher INR, PT, APPT, and D-dimer levels, combined with lower FIB, PLT, Hb, and HCT levels relative to those who survived (Table 1). By contrast, age, pupillary reactions, GCS at admission, head CT findings, and ISS were also significantly related to 3-month favorable outcomes (Table 2). Multiple injuries were not significantly related to 3-month mortality, although the combination of TBI with either chest or abdominal injuries was significantly associated with 3-month unfavorable outcomes. Patients with an unfavorable outcome exhibited significantly higher INR, PT, APPT, and D-dimer levels, combined with lower FIB, PLT, Hb, and HCT levels compared to those with a favorable outcome.

**Table 1:** Summary of patient characteristics and coagulation tests of the dead and surviving patients

	Died (%)	Survived (%)	Total (%)	*P* value^a^
*N*	198 (100.0%)	2372 (100.0%)	2570 (100.0%)	
Age (mean ± SD)	56.25 ± 17.81	47.90 ± 16.10	48.54 ± 16.38	< .001
Sex				.391
Male	154 (77.8%)	1780 (75.0%)	1934 (75.3%)	
Female	44 (22.2%)	592 (25.0%)	636 (24.7%)	
Mechanism of injury				
Traffic accident	119 (60.1%)	1346 (56.7%)	1465 (57.0%)	.090
Fall	20 (10.1%)	358 (15.1%)	378 (14.7%)	
Stumble	44 (22.2%)	408 (17.2%)	452 (17.6%)	
Blow to head	6 (3.0%)	104 (4.4%)	110 (4.3%)	
Others	9 (4.5%)	156 (6.6%)	165 (6.4%)	
Pupillary reactions				
Both reacting	103 (52.0%)	2145 (90.4%)	2248 (87.5%)	< .001
One reacting	50 (25.3%)	183 (7.7%)	233 (9.1%)	
None reacting	45 (22.7%)	44 (1.9%)	89 (3.5%)	
GCS at admission	6.73 ± 3.31	11.34 ± 3.49	10.98 ± 3.69	< .001
≤8	145 (73.3%)	586 (24.7%)	731 (28.5%)	< .001
9-12	36 (18.2%)	653 (27.5%)	689 (26.8%)	
13-15	17 (8.6%)	1133 (47.8%)	1150 (44.7%)	
CT findings				
IPH or brain contusion	162 (81.8%)	1642 (69.2%)	1804 (70.2%)	< .001
SDH	104 (52.5%)	684 (28.8%)	788 (30.7%)	< .001
EDH	32 (16.2%)	662 (27.9%)	694 (27.0%)	< .001
DAI	19 (9.2%)	52 (2.2%)	71 (2.8%)	< .001
Multiple body injuries	13 (6.6%)	134 (5.6%)	147 (5.7%)	.594
Face	1 (0.5%)	14 (0.6%)	15 (0.6%)	1.000
Thorax	11 (5.6%)	92 (3.9%)	103 (4.0%)	.333
Abdomen	2 (1.0%)	8 (0.3%)	10 (0.4%)	.385
Extremities	2 (1%)	35 (1.5%)	37 (1.4%)	.828
External	1 (0.5%)	3 (0.1%)	4 (0.2%)	.719
ISS^b^	22.33 ± 6.12	14.77 ± 7.02	15.35 ± 7.24	< .001
INR	1.16 ± 0.24	1.06 ± 0.13	1.07 ± 0.14	< .001
PT (s)	13.44 ± 2.71	12.161.55	12.26 ± 1.70	< .001
APTT (s)	31.19 ± 14.65	27.30 ± 7.20	27.60 ± 8.09	< .001
FIB (g/L)	2.06 ± 1.16	2.58 ± 1.13	2.54 ± 1.14	< .001
D-dimer (mg/L)	10.52 ± 14.67	6.90 ± 10.73	7.18 ± 11.12	< .001
PLT (× 10^9^/L)	156 ± 61	177 ± 67	176 ± 67	< .001
Hb (g/L)	124 ± 25	131 ± 21	131 ± 21	< .001
HCT (%)	36.44 ± 6.92	38.36 ± 5.69	38.21 ± 5.82	< .001

*GCS* Glasgow Coma Scale, *ISS* Injury Severity Score, *PLT* platelet, *INR* international normalized ratio, *PT* prothrombin, *APTT* activated partial thromboplastin time, *FIB* fibrinogen, *EDH* epidural hematoma, *SDH* subdural hematoma, *IPH* intraparenchymal hemorrhage, *DAI* diffuse axonal injury

^a^*P* values not adjusted for multiple comparisons. Statistical significance determined by chi-square test, Student’s *t* test or Mann-Whitney tests

^b^ISS = AIS1^2^ + AIS2^2^ + AIS3^2^, it is the sum of squares for the highest values in each of the three most severely injured body regions

**Table 2:** Summary of patient characteristics and coagulation tests of the favorable and unfavorable outcome patients

	Unfavorable outcome (%)	Favorable outcome (%)	Total (%)	*P* value^a^
*N*	802 (100.0%)	1768 (100.0%)	2570 (100.0%)	
Age (mean ± SD)	52.65 ± 16.20	46.68 ± 16.13	48.54 ± 16.38	< .001
Sex				.885
Male	605 (75.4%)	1329 (75.2%)	1934 (75.3%)	
Female	197 (24.6%)	439 (24.8%)	636 (24.7%)	
Mechanism of injury				
Traffic accident	469 (58.5%)	996 (56.3%)	1465 (57.0%)	.060
Fall	136 (17.0%)	242 (13.7%)	378 (14.7%)	
Stumble	132 (16.5%)	320 (18.1%)	452 (17.6%)	
Blow to head	21 (2.6%)	89 (5.0%)	110 (4.3%)	
Others	44 (5.5%)	121 (6.8%)	165 (6.4%)	
Pupillary reactions				
Both reacting	565 (70.4%)	1683 (95.2%)	2248 (87.5%)	< .001
One reacting	160 (20.0%)	73 (4.1%)	233 (9.1%)	
None reacting	77 (9.6%)	12 (0.7%)	89 (3.5%)	
GCS at admission	7.55 ± 3.01	12.54 ± 2.82	10.98 ± 3.69	< .001
≤8	523 (65.2%)	208 (11.8%)	731 (28.5%)	< .001
9–12	226 (28.2%)	463 (26.2%)	689 (26.8%)	
13–15	53 (6.6%)	1097 (62.0%)	1150 (44.7%)	
CT findings				
IPH or brain contusion	687 (85.7%)	1117 (63.2%)	1804 (60.2%)	< .001
SDH	361 (45.0%)	427 (24.2%)	788 (30.7%)	< .001
EDH	173 (21.6%)	521 (29.5%)	694 (27.0%)	< .001
DAI	50 (6.2%)	20 (1.2%)	71 (2.8%)	< .001
Multiple body injures	70 (8.7%)	77 (4.4%)	147 (5.7%)	< .001
Face	3 (0.4%)	2 (0.7%)	15 (0.6%)	.509
Thorax	57 (7.1%)	46 (2.6%)	103 (4.0%)	< .001
Abdomen	6 (0.7%)	4 (0.2%)	10 (0.4%)	.014
Extremities	16 (2%)	21 (1.2%)	37 (1.4%)	.158
External	3 (0.4%)	1 (0.1%)	4 (0.2%)	.176
ISS^b^	21.5 ± 6.4	12.57 ± 5.73	15.35 ± 7.24	< .001
INR	1.10 ± 0.16	1.06 ± 0.13	1.07 ± 0.14	< .001
PT(s)	12.59 ± 1.92	12.11 ± 1.57	12.26 ± 1.70	< .001
APTT(s)	28.50 ± 9.62	27.19 ± 7.26	27.60 ± 8.09	< .001
FIB(g/L)	2.45 ± 1.39	2.58 ± 1.01	2.54 ± 1.14	.016
D-dimer (mg/L)	10.97 ± 15.44	5.46 ± 7.89	7.18 ± 11.12	< .001
PLT (× 10^9^/L)	165 ± 70	181 ± 65	176 ± 67	< .001
Hb (g/L)	125 ± 24	133 ± 20	131 ± 21	< .001
HCT (%)	36.83 ± 6.62	38.83 ± 5.29	38.21 ± 5.82	< .001

*GCS* Glasgow Coma Scale, *ISS* Injury Severity Score, *PLT* platelet, *INR* international normalized ratio, *PT* prothrombin, *APTT* activated partial thromboplastin time, *FIB* fibrinogen, *EDH* epidural hematoma, *SDH* subdural hematoma, *IPH* intraparenchymal hemorrhage, *DAI* diffuse axonal injury

^a^*P* values not adjusted for multiple comparisons. Statistical significance determined by chi-square test, Student’s *t* test or Mann-Whitney tests

^b^ISS = AIS1^2^ + AIS2^2^ + AIS3^2^, it is the sum of squares for the highest values in each of the three most severely injured body regions

**Fig. 1:**
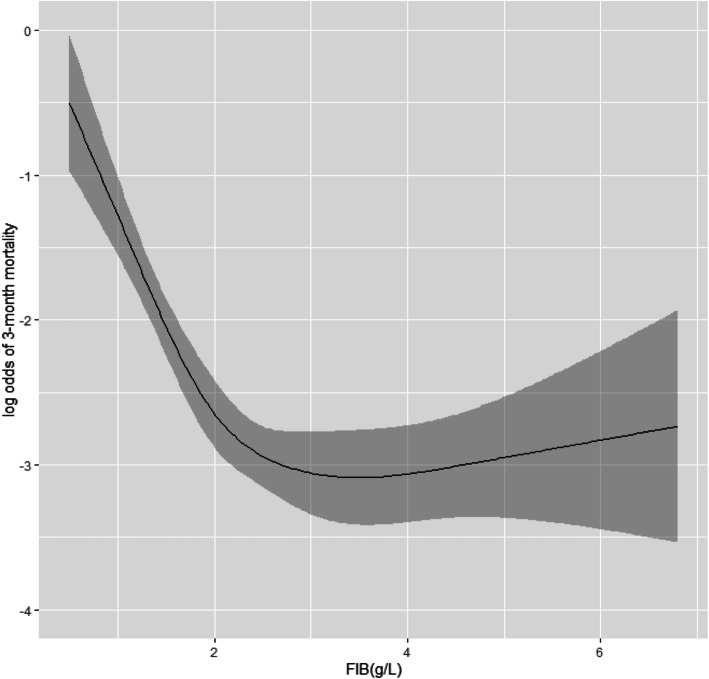
The shape of the relationship between the fibrinogen level at admission and the probability of 3-month mortality. When fibrinogen levels were < 2.0 g/L, mortality was inversely correlated with those levels. However, when they were > 2.0 g/L, the association between mortality and fibrinogen level was lost

The shape of the relationship between fibrinogen level and clinical outcomes was examined by univariate analyses with linear and cubic spline functions. The relationship between the fibrinogen level and the probability of favorable outcome and mortality was curvilinear, and the fitting degree of cubic spline functions was higher than that of a linear function. According to the curve correlation between fibrinogen levels and the probability of mortality, we found that when fibrinogen levels were < 2.0 g/L, mortality was inversely correlated with those levels. However, when they were > 2.0 g/L, the association between mortality and fibrinogen level was lost. Therefore, fibrinogen levels were divided into two subgroups of < 2.0 g/L and ≥ 2.0 g/L (Fig. 1). For patients with fibrinogen levels < 2.0 g/L, the levels were an independent prognostic factor of 3-month mortality in multivariate analyses (odds ratio [OR], 0.91; 95% confidence interval [CI], 0.89–0.93; *P* < .001). Age, GCS, pupillary reactions, EDH, and INR were also independent prognostic factors for mortality in this group. The risk of death in patients with fibrinogen levels < 2.0 g/L was 0.622 times higher (95% CI, 0.439–0.882; *P* = .008) than that in patients with fibrinogen levels ≥ 2.0 g/L (Table 3).

**Table 3:** Correlation between fibrinogen levels and mortality

Predictors	OR (95% CI)	*P* value^*a*^
**3-month mortality for patients with fibrinogen level < 2.0 g/L**		
Age	1.035 (1.019–1.051)	< .001
GCS	0.811 (0.739–0.891)	< .001
Pupillary reactions	2.240 (1.467–3.421)	< .001
EDH	0.280 (0.149–0.526)	< .001
INR	4.316 (1.556–11.975)	.005
FIB	0.522 (0.273–0.995)	.048
**3-month mortality for patients with fibrinogen level < 2.0 g/L vs. ≥ 2.0 g/L groups**		
AGE	1.035 (1.024–1.047)	< .001
GCS	0.760 (0.716–0.807)	< .001
Pupillary reactions	1.874 (1.404–2.500)	< .001
EDH	0.399 (0.253–0.630)	< .001
PT	1.215 (1.125–1.312)	< .001
FIBG^*b*^	0.622 (0.439–0.882)	.008

*GCS* Glasgow Coma Scale, *INR* international normalized ratio, *PT* prothrombin, *FIB* fibrinogen, *EDH* epidural hematoma, *SDH* subdural hematoma

^a^*P* values adjusted for multiple comparisons. Statistical significance determined by multiple logistic regression analysis

^b^FIBG means patients with fibrinogen level < 2.0 g/L or ≥ 2.0 g/L groups

**Fig. 2:**
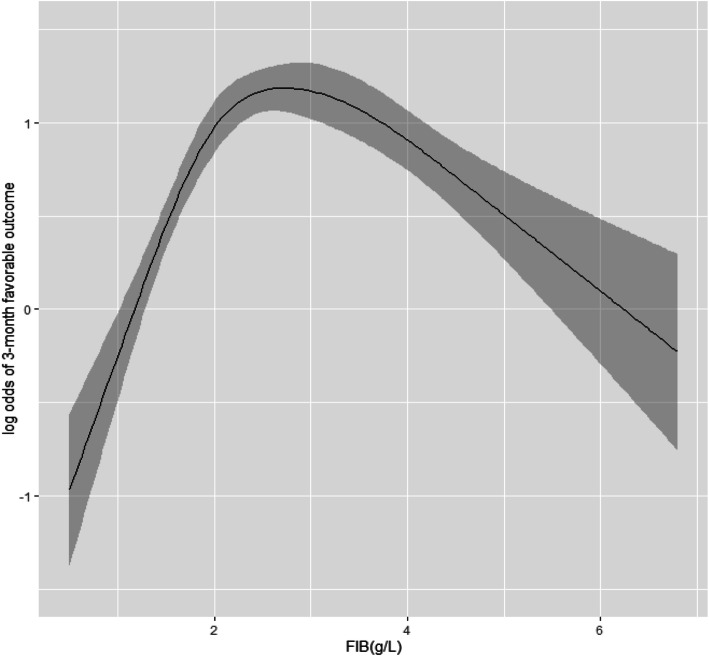
The shape of the relationship between the fibrinogen level at admission and the probability of 3-month favorable outcome. When the fibrinogen levels were < 2.5 g/L, the likelihood of favorable outcomes increased in association with fibrinogen level. This positive association was not seen in patients with fibrinogen levels between 2.5 and 3.0 g/L. Further elevation of fibrinogen to levels > 3.0 g/L revealed a decrease in favorable outcomes in association with increases in the levels

Further analyses of the correlation between fibrinogen levels and the probability of favorable outcomes showed that when the fibrinogen levels were < 2.5 g/L, the likelihood of favorable outcomes increased in association with fibrinogen level. This positive association was not seen in patients with fibrinogen levels between 2.5 and 3.0 g/L. Further elevation of fibrinogen to levels > 3.0g/L revealed a decrease in favorable outcomes in association with increases in the levels (Fig. 2). For patients with fibrinogen levels < 2.5 g/L, the levels were an independent prognostic factor for 3-month favorable outcomes (OR, 1.654; 95% CI, 1.186–2.306; *P* = .003). Age, GCS score, D-dimer, SDH, EDH, IPH, brain contusion, and thorax injury were also independent prognostic factors for favorable outcomes in this group. Similarly, for patients with fibrinogen levels > 3.0 g/L, multivariate analyses showed that the levels were also an independent prognostic factor for 3-month favorable outcomes (OR, 0.771; 95% CI, 0.607–0.979; *P* = 0.033), with GCS, D-dimer, IPH, and brain contusions also independent prognostic factors for favorable outcomes in this group (Table 4).

**Table 4:** Correlation between fibrinogen levels and the probability of favorable outcomes

Predictors	OR (95% CI)	*P* value^a^
**3-month favorable outcome for patients with fibrinogen level < 2.5 g/L**		
AGE	0.965 (0.955–0.975)	< .001
GCS	1.624 (1.539–1.714)	< .001
SDH	0.601 (0.440–0.821)	.001
EDH	1.615 (1.124–2.321)	.010
IPH or brain contusion	0.448 (0.302–0.665)	< .001
D-dimer	0.975 (0.964–0.986)	< .001
FIB	1.654 (1.186–2.306)	.003
Thorax injury	0.509 (0.277–0.934)	.029
**3-month favorable outcome for patients with fibrinogen level > 3.0 g/L**		
Predictors	OR (95% CI)	*P* value^a^
GCS	1.555 (1.450–1.668)	< .001
IPH or brain contusion	0.446 (0.246–0.808)	.008
D-dimer	0.932 (0.899–0.966)	< .001
FIB	0.771 (0.607–0.979)	.033

*GCS* Glasgow Coma Scale, *FIB* fibrinogen, *EDH* epidural hematoma, *SDH* subdural hematoma, *IPH* intraparenchymal hemorrhage

^a^*P* values adjusted for multiple comparisons. Statistical significance determined by multiple logistic regression analysis

## Discussion

Hypofibrinogenemia is an important indicator of poor prognosis in trauma patients [18]. European guidelines on management of major bleeding and coagulopathy following trauma recommend the use of fibrinogen concentrate or cryoprecipitate if major bleeding is accompanied by hypofibrinogenemia (viscoelastic signs of a functional fibrinogen deficit or a plasma fibrinogen level ≤ 1.5 g/L) [14]. However, less is known regarding the association between fibrinogen concentration and prognosis in patients with TBI. Therefore, we conducted a retrospective study examining patients over the course of 15 years to better assess the relationship between fibrinogen levels at the time of admission and clinical outcomes in patients with TBI. We found that hypofibrinogenemia is very common in patients with TBI, with 15.7% of all patients exhibiting fibrinogen levels < 1.5 g/L, and 38.6% of patients of patients with levels < 2.0 g/L.

Our analyses showed that the relationship between fibrinogen levels at the time of admission and the probability of favorable outcome and mortality was not nonlinear but rather curvilinear. When fibrinogen was < 2.0 g/L, these levels were an independent prognostic factor for 3-month mortality. However, for patients with fibrinogen levels < 2.5 g/L, multivariate analyses showed that the levels were instead an independent prognostic factor for 3-month favorable outcomes, with the likelihood of a favorable outcome increasing in association with an increase in level.

In addition to showing that low fibrinogen levels increase the risk of poor prognosis, we also provide evidence that a high fibrinogen level itself is a risk factor for poor prognosis. For patients with fibrinogen levels > 3.0 g/L, multivariate analyses showed that the levels were an independent prognostic factor for 3-month favorable outcomes. The likelihood of a favorable outcome decreased in conjunction with increases in fibrinogen levels. One possible explanation for the increase in unfavorable outcomes in the high fibrinogen group may be due to the role of fibrinogen as an acute-phase protein, indicating the presence of inflammatory diseases, such as atherosclerosis [18, 19]. In addition, TBI can damage the blood-brain barrier (BBB), causing blood components to extravasate into the brain parenchyma. Studies in different animal models of TBI have demonstrated both acute and delayed BBB damage [20, 21]. Secreted fibrinogen is transformed into fibrin, and releases reactive oxygen species, leading to activation of M1 macrophages and microglia, which may contribute to nerve damage [22, 23]. In TBI models, damaged axons are an important source of fibrinogen leakage. This localized increase in fibrinogen levels results in microglial cell clustering around damaged blood vessels, which in turn fuels a sustained acute microglial response and induces microglial cells to release reactive oxygen species, thereby causing axonal injury [24, 25]. These findings suggest that plasma levels of fibrinogen should be dynamically monitored in TBI patients prior to FIB supplementation to avoid excessive levels.

Age, GCS, pupillary reactions, EDH, and INR were all independent prognostic factors for mortality in patients with fibrinogen levels < 2.0 g/L. By contrast, age, GCS score, D-dimer, SDH, EDH, IPH, brain contusion, and thorax injury were all independent prognostic factors for favorable outcomes in patients with fibrinogen levels < 2.5 g/L, while GCS, D-dimer, IPH, and brain contusion were independent prognostic factors for favorable outcome in patients with levels > 3.0 g/L. These observations are consistent with previous reports that have indicate that ISS score is an indicator of clinical outcomes in patients with severe trauma. Floccard et al. also reported that FIB levels in trauma patients at the time of admission could be affected by ISS [26]. In our study, logistic regression analyses demonstrated no significant association between ISS and clinical outcomes in TBI patients. However, multivariate regression analyses did identify significant correlations, with strong associations between thoracic injury, as assessed by AIS score, and clinical outcomes in TBI patients with fibrinogen levels < 2.5 g/L. TBI patients who also suffered thoracic injury exhibited a 1.96-fold increase in unfavorable outcomes compared to patients with no such injury. This result is consistent with our previous findings that thoracic injury is frequent in patients with TBI and is closely related to TBI outcomes. Moreover, we found that thoracic injury remains the most frequent injury, accounting for 70% of all secondary injuries in TBI patients.

In traumatic hemorrhage, the amplifying mechanism in the early stage of coagulation causes a surge in thrombin production [27]. Fibrinogen is converted into fibrin under the action of thrombin, which leads to the consumption of fibrinogen [28]. Studies in tissue injury demonstrate that endothelial injury accelerated fibrinogen generation and triggered hyperfibrinolysis [29]. Thrombin generation response to trauma combined the effects of hyperfibrinolysis further aggravate fibrinogen loss [30]. This may also explain why nearly 40% of TBI patients suffer from hypofibrinogenemia. However, the relationship between thrombin and fibrinogen in consumptive coagulopathy needs to be further studied.

Utilizing serum biomarker to early diagnose hypofibrinogenemia has the potential to overwhelm the adverse prognosis in TBI patients. Serum fibrinogen concentration is the most direct evidence of fibrinogen deficiency in patients with TBI. Additional biomarkers included plasma levels of antithrombin, protein C, APTT, and head CT scan [31]. Our data demonstrate that fibrinogen might be a valuable serum biomarker to differentiate TBI patients with hypofibrinogenemia from acute traumatic coagulopathy, as well as to potentially guideline fibrinogen supplementation during acute trauma care.

Some limitations of our study should be acknowledged. All analyses were performed retrospectively. While the large sample size was a strength, all subjects were recruited from a single neurotrauma center and the time of patient enrollment became relatively large (14 years). A well-designed multicenter, prospective, randomized clinical trial is needed to achieve more accurate results. Second, this study excluded patients who received blood product transfusions prior to admission but did not take into account the dilutional effect of the volume of the fluid given before arriving at the emergency room. To reduce this effect, the results of the first FIB test at the time of admission were studied. However, it is difficult to ensure standardized timing for the collection of blood samples, which may introduce some variability into the first fibrinogen concentration. Third, we only studied the relationship between fibrinogen at the time of admission and prognosis. Whether fibrinogen was supplemented during hospitalization or perioperative period and relevant data were not recorded. Finally, the Marshall CT scan scores of patients involved in our study are extremely hard to obtain because of the long period of patient enrollment time.

## Conclusions

In conclusion, we observed fibrinogen concentrations < 2 g/L in 38.6% of TBI patients at the time of admission, with this level strongly related to an increased in-hospital mortality. Comparisons of fibrinogen levels and clinical outcomes suggested that maintaining fibrinogen levels between 2.5 and 3.0 g/L may be the best way to improve prognosis, although further prospective studies are needed to confirm these observations.

## Data Availability

The datasets used or analyzed during the current study are available from the corresponding author on reasonable request.

## Abbreviations

TBI: Traumatic brain injury
GCS: Glasgow Coma Scale
GOS: Glasgow Outcome Score
ISS: Injury Severity Score
PLT: Platelet
INR: International normalized ratio
PT: Prothrombin
APTT: Activated partial thromboplastin time
Hb: Hemoglobin
HCT: Hematocrit
FIB: Fibrinogen
EDH: Epidural hematoma
SDH: Subdural hematoma
IPH: Intraparenchymal hemorrhage
DAI: Diffuse axonal injury
